# Novel Bi-UWB on-Chip Antenna for Wireless NoC

**DOI:** 10.3390/mi13020231

**Published:** 2022-01-30

**Authors:** Hafedh Ibrahim Gaha, Moez Balti

**Affiliations:** 1 Telecommunication Department, Higher Institute of Technological Studies in Communications of Tunis (Iset’Com), El Ghazala Technopark, Ariana 2088, Tunisia; moez.balti@edu.isetcom.tn; 2 Electronic Systems and Communications Networks Laboratory (SERCOM), Polytechnic School of Tunisia, Carthage University, Tunis 1080, Tunisia

**Keywords:** wireless communication (WiNoC), on-chip antenna, multi-band, frequency assignment

## Abstract

Communication between on-chip cores is a challenging issue for high-performance network-on-chip (NoC) design. Wireless NoC (WiNoC) represents an alternative design for planar wired interconnects, aiming to reduce latency and improve bandwidth. In this paper, a novel on-chip fractal antenna is designed and characterized. In order to disseminate interference affecting NoC performance in order to enhance on-chip quality of service (QoS), a set of exclusive sub-channels are assigned to each antenna. The proposed antenna has two wide bands (bi-WB)—$B_{1}$ and $B_{2}$, of (63–78) GHz and (101–157) GHz, respectively. The multi-band antenna allows different channel allocations for on-chip core communications. This WiNoC design exhibits improved performance, due to its enhanced antenna bandwidth and the benefit provided by the developed algorithm that can scan and compare to assign the best (upload or download) sub-channels to each antenna.

## 1. Introduction

Emerging applications such as bio-telemetry, GPS, and RFIC for wrist-wearable communication applications, wireless clock distribution, IoT/biomedical, wireless power transfer, wirelessly powered dielectric sensors, sensor networks and wireless tagging, wireless network-on-chip (WNoC) systems and chip-to-chip wireless communication systems, have motivated innovations in wireless transceiver systems, components, architectures, and technologies [1]. In these applications, wireless communication with a wide bandwidth is essential in order to achieve a higher data rate. System-on-chip (SoC) systems based on a multi-processor (MPSoC) architecture with a wide variety of heterogeneous intellectual property (IP) blocks ensure that on-chip communication plays a key role in determining the reliability, performance, area, and power consumption of these devices. Developers of network-on-chip (NoC) architectures have suggested shifting the bus interconnections [2] to wireless communications, since MPSoC systems require such changes. Wireless NoC present the potential for scalable interconnect architectures with a reduced latency for next-generation NoCs; therefore, the wireless-NoC topology can be totally different from that of a traditional NoCs. Recent research has implemented wireless-NoC (WiNoC)-based antennas [3,4] to provide a scalable and flexible on-chip communication infrastructure. On-chip antennas have several advantages, which lead to low signal losses and higher integration levels, as well as improved latency and broadcasting capabilities. In the literature, on-chip antennas operate on a single wireless channel based on the resonant frequency.

The advantage of ultra-wide-band (UWB) antennas is their quasi-omnidirectional characteristics and their good radiation [5]. Many mictrostrip-fed antenna designs have revealed acceptable behavior when targeting the use of the free licence band from 3.1 to 10.6 GHz, especially when they have circular and elliptical coplanar waveguide (CPW)-fed slots [6]. Reference [7] showed how frequency-selective surface (FSS) techniques enhance antenna gain for UWB antennas. This technique was deployed to boost the performance of UWB antennas and to increase the gain by using a circular polarized (CP) antenna. Reference [8] studied and compared a V-shaped UWB monopole antenna and dual-band UWB notch antenna to the previous literature. They observed less group delay, a linear phase, stable radiation patterns, and dual notch bands, making their system feasible for use in UWB applications.

We were inspired by the existing literature to design a novel Bi-WB on-chip antenna for wireless NoC applications. As the number of on-chip intellectual properties (IPs) continues to increase, wireless communication technology channels are becoming limited [4], particularly for simultaneous wireless communication. A recent work proposed a multi-channel wireless NoC based on traditional NoC topologies [9]. Despite challenges owing to the on-chip antenna design, this paper suggests a novel on-chip antenna with wireless bi-wide-band frequencies. Multiple wireless channels are created by splitting the two proposed antenna bands into channels. Each channel is assigned exclusively to the communication between each pair of antennas.

This work is structured as follows. Section 2 (Wireless-Based NoC Topology) presents the wireless topology. In Section 3 (Wireless Channel), the channel characteristics and indoor model are provided for no-loss environment conditions. A novel on-chip antenna (NoChA) is presented, characterized and a description of the design steps is provided in Section 4 (On-Chip Antenna). Then, a brief comparison with some reference antennas is presented. Section 5 (Assignment of Frequencies) describes the frequency channel assignment process. Section 6 highlights the principal conclusions and perspectives. Eventually, the study of (4×4) antenna-dispositions is presented in Appendix A with three subsections detailing how we chose the best arrangement among all the possible cases investigated.

## 2. Wireless-Based NoC Topology

On-chip network topology is a key factor that impacts and determines the quality of service (QoS) of the designed network, as the network’s ability to efficiently communicate depends on its topology [10]. The on-chip hops count for a packet to traverse an NoC between the source and destination, and IP cores impact the topology design, which affects network energy consumption and latency [11,12,13]. NoC design needs to take into account some metrics, such as latency and power consumption, to provide high performance.

### 2.1. Hybrid Topology

**Figure 1:**
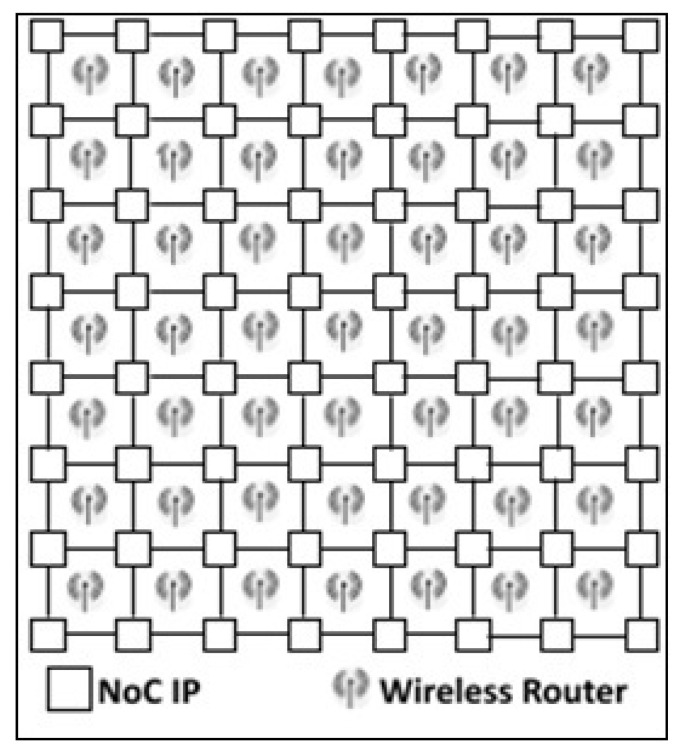
WiNoC hybrid topology based on wireless hubs.

A hybrid NoC consists of a two-level network. One level is a wired links network, which is intended for cases in which there is a reliably short-distance between the neighboring on-chip nodes, whereas the other level is a wireless link for distant communications, which can reduce latency and power efficiency, owing to single-hop communication between far IP cores, and which can enhance wireless bandwidth, limited by physical constraints for wired links. Figure 1 illustrates a generic example of an 8 × 8 2D mesh WiNoC topology based on 4 × 4 wireless hubs with wide-band antennas [14]. Each wireless hub is working as a router that allows single-hop communication between far-IP cores.

### 2.2. Pure Wireless Topology

**Figure 2:**
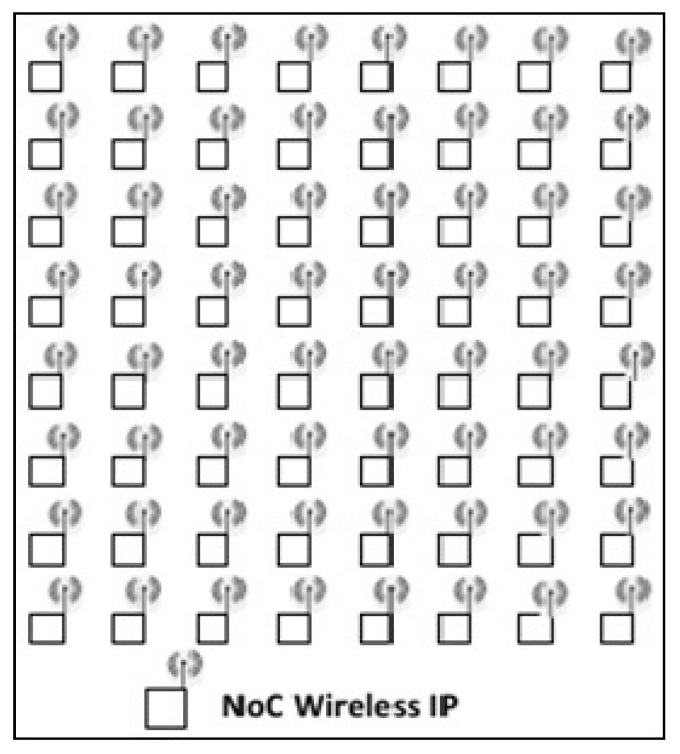
Pure WiNoC topology based on wireless hubs.

Despite the domination of hybrid topologies in actual WiNoC design, the design of pure WiNoCs seems to be an open issue in the proposal of some novel on-chip antennas. This new on-chip interconnection network, in which all the wired links should be replaced by wireless links (as depicted in Figure 2), provides high-bandwidth benefits, a low transmission latency, less power dissipations, and flexible topology configurations [15].

In both hybrid and pure wireless systems, the two crucial elements of the on-chip wireless hub are the antenna and the transceiver; their features are discussed in Section 4. However, the advantage of wireless links is their low latency over long distances and their disadvantage is the area and power overhead caused by the wireless transceiver. Thus, the prevailing design uses a hybrid wireless network, as mentioned in this paper. However, this article focuses on a purely wireless 4×4 network, in which the wired link is completely abandoned. Short-range wireless links have no advantage over wired links, but instead have a huge disadvantage in terms of reliability and the power consumption overhead. In this paper, hundreds of wireless links in different frequency bands are organized through spectral channels, but the overheads of wireless links are completely the work of many other researchers, who are working to overcome such critical issues [16,17,18,19]. The feasibility of this antenna design may be deployed in the context of a hybrid wireless network with an attempt to reach a purely wireless topology.

## 3. Wireless Channel

On-chip wireless channel characteristics largely govern the performance and bandwidth efficiency of wireless communication systems. Since the channel condition does not change due to the immobility of on-chip cores, the channel tends to be static. Furthermore, since on-chip communication occurs in a completely enclosed environment, the power azimuth spectrum will be uniform.

### Communication Model

High performance is becoming a complicated goal across systems on chip (SoCs) as the number of data channels scales up across all on-chip communication NoCs; thus, channel performance becomes more difficult to predict. In this work, we consider the indoor two-ray channel model. The two-ray model has been used to explain the observed path loss and provides a simple way to predict the received power [20,21]. It consists of two rays with the same power. The first ray is for a direct path with zero delay and a null *τ* = 0, whereas the second ray is for a longer path, considered after reflection, with a delay *τ* > 0, which is the parameter that determines the model’s characteristics as given in [22,23,24]. The two-ray model is a suitable choice for millimeter waves (mmWave), and especially for channels with reflections [25]. For the on-chip application in which the channel distance is about a few millimeters, the power magnitude of the second path could be equal to that of the first power path, which makes this model acceptable and useful in practice. To predict the received signal in an environment considered without any obstacle between the transmitter and receiver, and a system hardware assumed without loss, the free-space propagation model presented by Friis [26] is used to express the received power $P_{r}$ at distance *d*, as shown in Equation (1).

(1) $$P_{r}\left(d\right)=\frac{P_{t}G_{r}G_{t}\lambda^{2}}{{\left(4\pi d\right)}^{2}}$$

where $P_{t}$ represents the transmitted power (watts); $G_{t}$ and $G_{r}$ are the transmitted gain and the received gain, respectively; $P_{r}$ is received power at distance *d*; and λ is the wavelength (m).

## 4. On-Chip Antenna

### 4.1. Antenna Structure

The introduction of a wireless interconnect introduces the opportunity for a high data rate, low power consumption, and low-cost implementation for on-chip millimeter-range communication. The on-chip antenna must meet the following conditions. It must be sufficiently small, highly efficient [27,28], and show the best power gain at the minimal area overhead. The on-chip fractal antenna is designed to provide multiband wireless communication. Each band is subdivided into channels allocated to on-chip IP core communications [29]. This allows the most appropriate assignment of spectral channels to each on-chip IP core, hence making the antenna a suitable candidate for *WiNoC* applications. Multi-channel techniques are easily adopted in *WiNoC* designs for interference dissemination and simultaneous communication, which improve the network throughput and latency. Recently, more on-chip antennas operating in the millimeter wave range have been proposed to enable wireless interconnection [30,31,32]. The antennas’ sizes should be approximately equal to the wavelength, on the order of a few millimeters.

**Figure 3:**
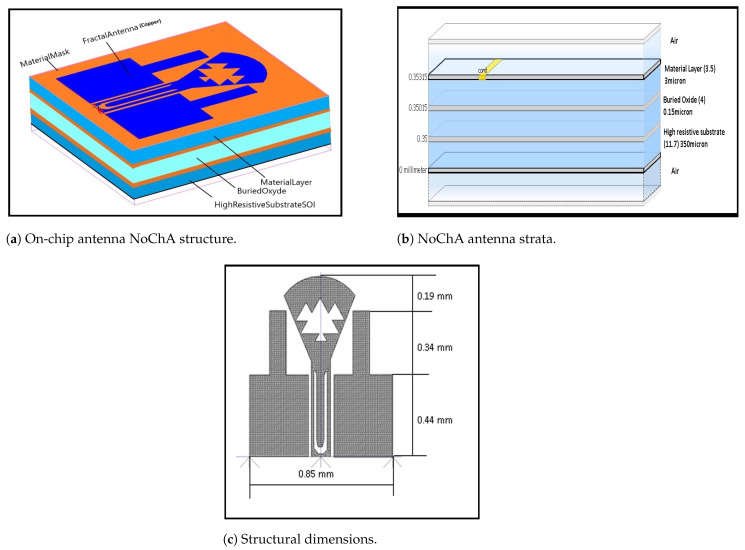
On-chip antenna NoChA: dimensions and structure.

The antenna size is reduced accordingly with the transmission frequency increase. Since the on-chip IP-cores are about 5 to 25 mm^2^, the designed antenna should occupy less than 1 mm^2^. Figure 3a represents an antenna with the different deployed layers where the conducting element is covered by air. Figure 3c provides the proposed antenna dimensions having a size of about [0.85 mm; 0.94 mm]. In Section 4.2, we explain how we have chosen these sizes. The fractal antenna technology [11] consists of a copper layer printed on a multi-layer substrate that includes a dielectric layer with a relative dielectric permittivity $\epsilon_{r}=3.5$, a buried oxide layer with $\epsilon_{r}=4$, a high-resistivity (HR) layer with $\epsilon_{r}=11.7$, as shown in Figure 3b. The deployment of such a HR layer is well justified by its lower dielectric losses and its high capacity for antenna miniaturization.

Moreover, antenna efficiency $\eta_{a}$ depends upon Equation (2) where $R_{ri}$ and $R_{losses}$ indicate the resistance of radiation and losses, respectively; such losses are due to $R_{dielectric}$, $R_{Ohmic}$, and $R_{ground}$. We mention here the dielectric resistance, which helps designers to choose their dielectrics carefully with the minimum of losses; especially, tg($\delta_{e}$), the electric loss tangent, and tg($\delta_{m}$), the magnetic loss tangent, expressed in Equation (3) where complex permittivity and permeability are expressed as in Equation (4).

(2) $$\eta_{a}=\frac{R_{ri}}{R_{ri}+R_{losses}},\;R_{losses}=R_{dielectric}+R_{Ohmic}+R_{ground}.$$

The tangent of electric and magnetic losses are:

(3) $$tg\left(\delta_{e}\right)=\frac{\epsilon^{\prime \prime }}{\epsilon^{\prime }},\;tg\left(\delta_{m}\right)=\frac{\mu^{\prime \prime }}{\mu^{\prime }},$$

as:

(4) $$\epsilon =\epsilon^{\prime }-i\times \epsilon^{\prime \prime },\;\mu =\mu^{\prime }-i\times \mu^{\prime \prime }.$$

Furthermore, in [33] the author concludes his HR dielectric deployment justification on page 231 to page 238, and describes many applications deploying HR layers, as explained, for example, on page 235. The total chip height is approximately 359.15 µm. The antenna provides circular polarizations based upon dual port excitation. U-slots are adjusted to provide multiband frequency. Nowadays, silicon (Si especially silicon-germanium (SiGe) integrated in BiPolar CMOS (SiGe BiCMOS)) based technologies have become an attractive choice for the on-chip antennas design due to its reduced cost, small chip-size, low power and high integration level. The low resistivity and high permittivity ($\epsilon_{r}=11.7$) values of Si-based technologies are unable to provide on-chip antennas with good performance. Another technology, such as silicon on insulator (SOI), has been proposed for on-chip antenna design due to its high resistivity substrate, in order to improve the device’s performance.

### 4.2. Antenna Design Steps

**Figure 4:**
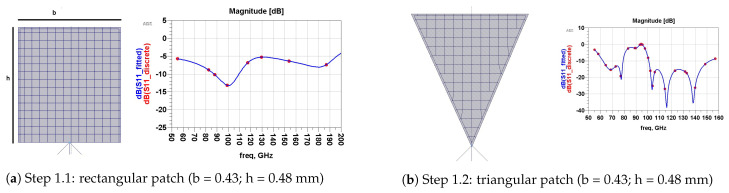
Step 1: simple view.

**Figure 5:**
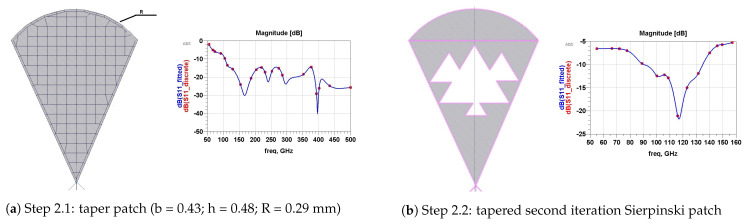
Step 2: ultra-wide and Sierpinski second iteration.

**Figure 6:**
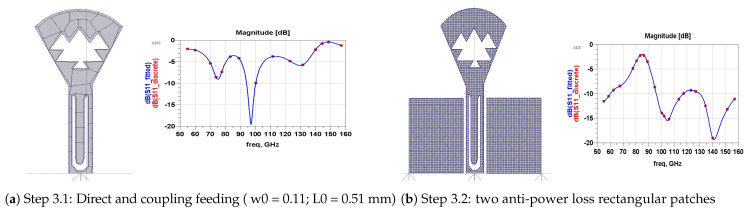
Step 3: Simple coupling feeding.

**Figure 7:**
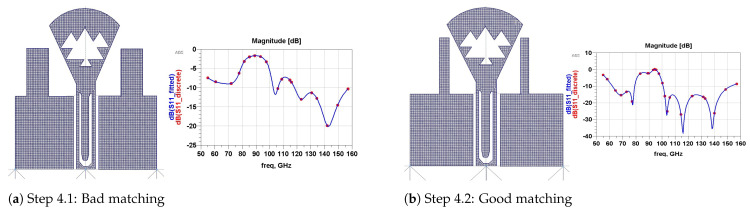
Step 4: Second pair of rectangular patches.

We designed our proposed antenna in four steps, as illustrated in Figure 4, Figure 5, Figure 6 and Figure 7. Each step is indicated via its $S_{11}$ value.The first step is shown in Figure 4. We intend the proposed antenna to operate in the range of (50–200) GHz; therefore, we began simulating a rectangular-patch antenna with *h* as the height and *b* as the width. These dimensions are approximated as follows (Figure 4a):

$$\left.\begin{array}{cc} \frac{\lambda }{4}\approx h\approx b & \\ \frac{\lambda }{4}=\frac{c}{4f} & =\frac{3\times 10^{8}}{4\left[50,200\right]\times 10^{9}}=\left[375,1500\right]\mu m \end{array}\right\}\Rightarrow \left(h,b\right)\approx \left[480,430\right]\mu m$$

With the same dimension (h,b), Figure 4b represents a triangular-patch antenna that would resonate into unstable wide-bands. In Figure 5, (step 2) we added a part of a disk with a radius R=270 µm and a cord *b*. This renders the wide aspect of the antenna. In Figure 5b, we introduced triangular Sierpinski hollows in its second iteration. This produces multiple contiguous wider sub-bands. Figure 6 (step 3) explains how we designed antenna matching. Figure 6a represents the two forms of feeding used: directly and by coupling a U feeder enclosed in a rectangle ($w_{0}=110$, $L_{0}=510$) µm. In Figure 6b, we optimized our antenna matching by adding two rectangular patches ($w_{1}=348$, $L_{1}=438$) µm. This filters some frequencies and limits power losses.

Figure 7 (step 4) demonstrates how we have limited more power losses by adding two other rectangular patches with different dimensions ($w_{2}=133$, $L_{2}=283$ µm (Figure 7a) and $w_{2}=47$, $L_{2}=340$ µm (Figure 7b)).

All these steps helped us to obtain an ameliorated version of our proposed antenna, with the following dimensions: b=426, h=473, R=275 µm; $w_{0}=118$, $L_{0}=512$ µm; $w_{1}=350$, $L_{1}=442$ µm; and $w_{2}=113$, $L_{2}=346$ µm. The Sierpinski hollows are formed of four triangles, as shown in Figure 5b. The biggest one has a height and base of 188 and 233 µm, respectively; the smallest one has a height and base of 50 and 70 µm; and the two others have heights and bases of 95 and 125 µm.

### 4.3. Antenna Characterization

**Figure 8:**
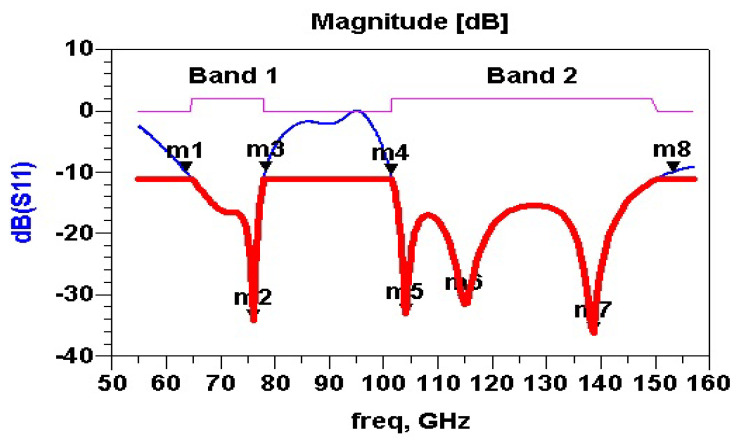
$S_{11}$(dB): Reflection coefficient of NoChA.

Figure 8 shows simulated $S_{11}$ parameters of the proposed NoChA fractal antenna. According to the −10 dB threshold (1/10 losses allowed), we distinguish two frequency bands as shown in Table 1—$B_{1}$(m1, m3) and $B_{2}$(m4, m8). However, when losses are required to be 1/100, the threshold must be less to −20 dB. Therefore, four bands are provided by our proposed antenna, centered around m2, m5, m6, and m7. The transmission coefficient $S_{ij}$ will be considered in antennae networks treated via IP coupling in Section 5. Table 1 gives the $S_{11}$ values for eight critical frequencies from Figure 8.

Herein we provide the fractional frequency band ratio FBR, which is the frequency band width FBW, expressed as a percentage. In our bi-band antenna, for each band, we associate the $F_{H}$ (highest frequency), $F_{L}$ (lowest frequency), $F_{c}$ (center frequency), and FBR as follows:

$$FBR=\frac{\left(F_{H}-F_{L}\right)}{F_{c}}\times 100\left(\%\right)=2\frac{\left(F_{H}-F_{L}\right)}{\left(F_{H}+F_{L}\right)}\times 100\left(\%\right)=\left\{\begin{array}{cc} FBR_{1}= & \frac{78.32-63.26}{70.79}\times 100=21.27\%\\ FBR_{2}= & \frac{157.0-101.2}{129.1}\times 100=43.22\%. \end{array}\right.$$

One can note that the higher the percentage, the wider the bandwidth. Wide-band antennas typically have an FBR of around 20%, whereas those with an FBR around 45% are considered UWB antennas.

The efficiency is given by $\eta =\frac{G}{D}$. Table 2 shows the antenna parameters depending on central frequencies, where the proposed antenna directivity is considerable but efficiency remains only acceptable and may be improved. *G* is the antenna gain given by Equation (5), and D is the directivity. This allows for deployment of the antenna even in a laminated on-chip structure (multi-layered).

**Figure 9:**
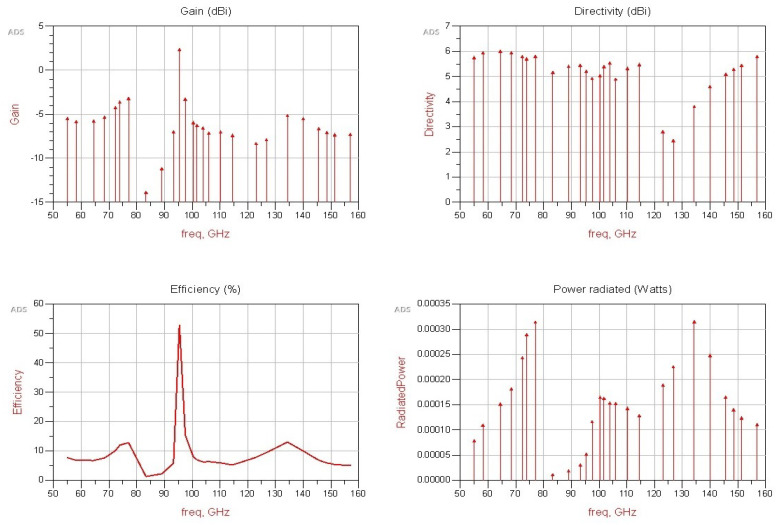
Gain, directivity, efficiency, and power radiated vs. frequency.

**Figure 10:**
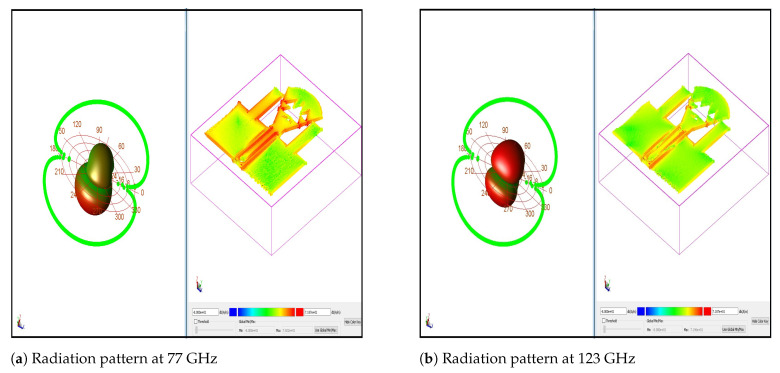
Radiation pattern at 77 GHz and 123 GHz (cut on ϕ=0) and the current density scaled from −80 dB (A/m) (blue) to 71.8 dB (A/m) (red).

**Figure 11:**
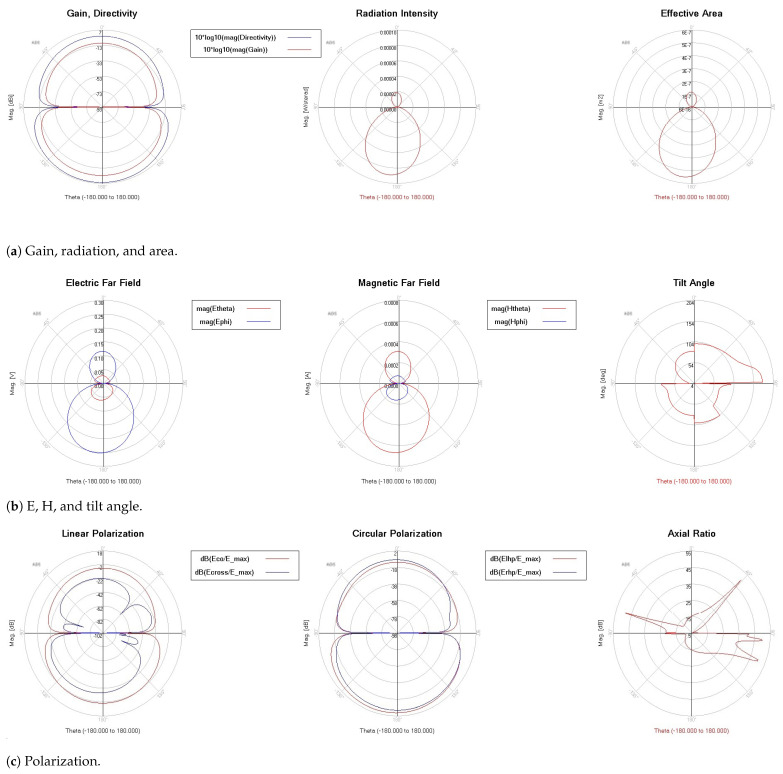
NoChA parameters at 77 GHz.

**Figure 12:**
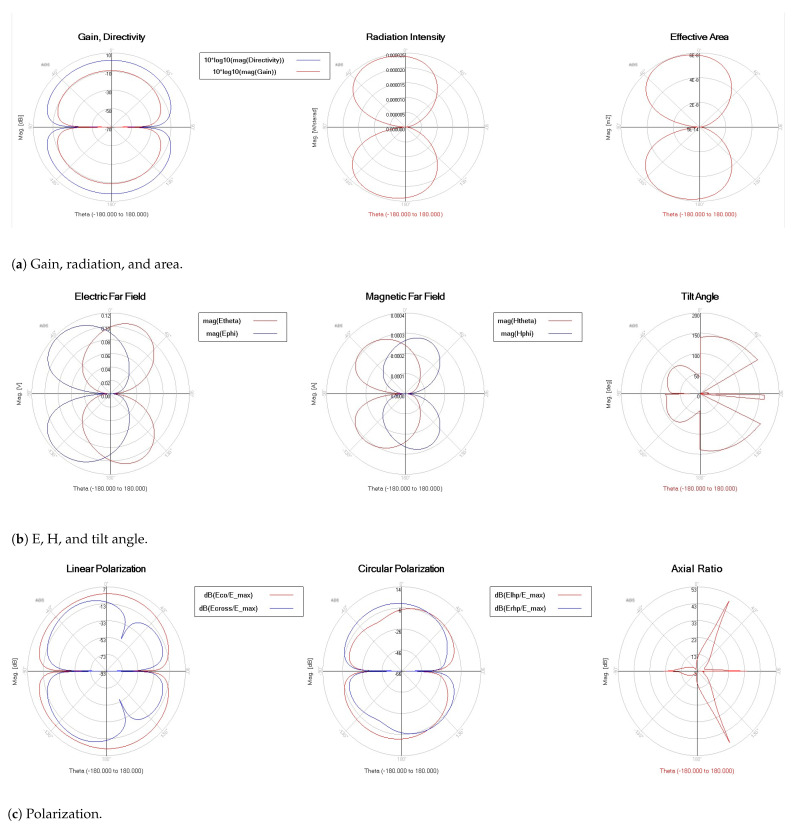
NoChA parameters at 123 GHz.

Figure 9 indicates the gain, directivity, efficiency, and radiated power. The curve (efficiency versus frequency) shows that the best efficiency is achieved at 95 GHz. The directivity is always in the range of 4–6 dBi for all the frequencies, except between 120 GHz and 130 GHz. However, radiated power (Watts) is sufficiently high, except between 80 GHz and 94 GHz, where gain and efficiency have the lowest values. Thus, gain is directly affected, and the best gain value is achieved when the efficiency has the highest value. Radiation patterns, as illustrated in Figure 10, prove how omni-directional the radiations are in several bands. In Figure 10b, we note that the radiation pattern at 123 GHz is better than that in Figure 10a at 77 GHz. We added the current density in two cases of f=77 GHz and f=123 GHz. We want to prove that the proposed antenna NoChA is radiating sufficiently to reach its neighbors, as we need it to operate in the NoC 4×4. Figure 11 and Figure 12 show the parameters of the proposed antenna in 77 GHz and 123 GHz, respectively, chosen from the bi-bands $B_{1}$ and $B_{2}$.

Table 3 provides a performance comparison of our work vs. those in [34,35,36,37,38,39]. Antenna types are mentioned in the last column, and the sizes are presented in the second column, showing that our antenna is not greater than 1 mm^2^, and our bi-wide-band antenna operates on two wide bands, whereas the other antennas use only one band. The best frequencies (according to $S_{11}$) are presented in the fifth column. The bandwidth of each case is shown in the third column. We can consider our design satisfactory, due to its gain (shown in the fourth column) and its two UWB bands.

## 5. Assignment of Frequencies

**Figure 13:**
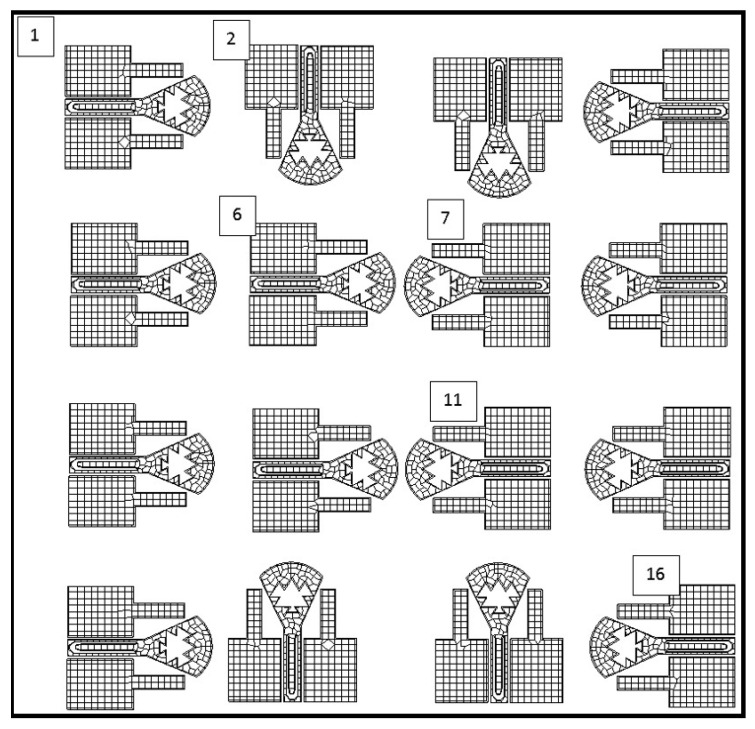
Disposition of 4×4 IP-Antennas from 1 to 16.

In this section, we identify the best antenna layout, providing the best gains $G_{ij}$. We consider a wireless based-NoC topology of 4×4 on-chip cores, as depicted in Figure 13. To achieve this optimal layout of the antennas, an algorithm has been proposed. The algorithm scans all possible layouts randomly by calculating the parameters *S*. The best parameters indicate the best layout.

After calculating the $S_{ii}$ and the transmission coefficient $S_{ij}$ of on-chip antennas, the assignment of channel frequencies would respect the following constraints:

- Rule 1: $S_{ii}$(dB) ≤ threshold, for example, −10 dB, as we do not tolerate the tenth losing power. (giving $B_{ik}$ and $Ch_{ikp}$) and k represents the indexes of the appropriate bands. Frequency bands $B_{ik}$ are deduced from Figure 8 as follows: $B_{i1}$ (m1, m3) and $B_{i2}$ (m4, m8). Markers’ values are indicated in Table 1.
- Rule 2: $S_{ij}$(dB) ≥ (2/3 of max($S_{ij}$) or more than −20 dB (giving $Bt_{ij,k}$ and $Cht_{ij,k,p}$).
- Rule 3: One or more bands $B_{ik}$ are allocated to the $IP_{A_{i}}$, where Rule 1 is respected.
- Rule 4: Each *B_ik_* is subdivided into one or more channels Chikp, where p represents the indexes of the appropriate channels, referring to *B_ik_*.

where *i* is the antenna index from 1 to 16, *k* is the frequency band index (1,2), and p is the channel index. The frequency assignment process, respecting the above rules, should eliminate frequency interferences. The transmit gain $G_{ij}$ from antennas $A_{j}$ to $A_{i}$ is given by Equation (5):

(5) $$G_{ij}=|S_{ij}{|}^{2}/((1-|S_{ii}{|}^{2})(1-|S_{jj}{|}^{2}))$$

**Figure 14:**
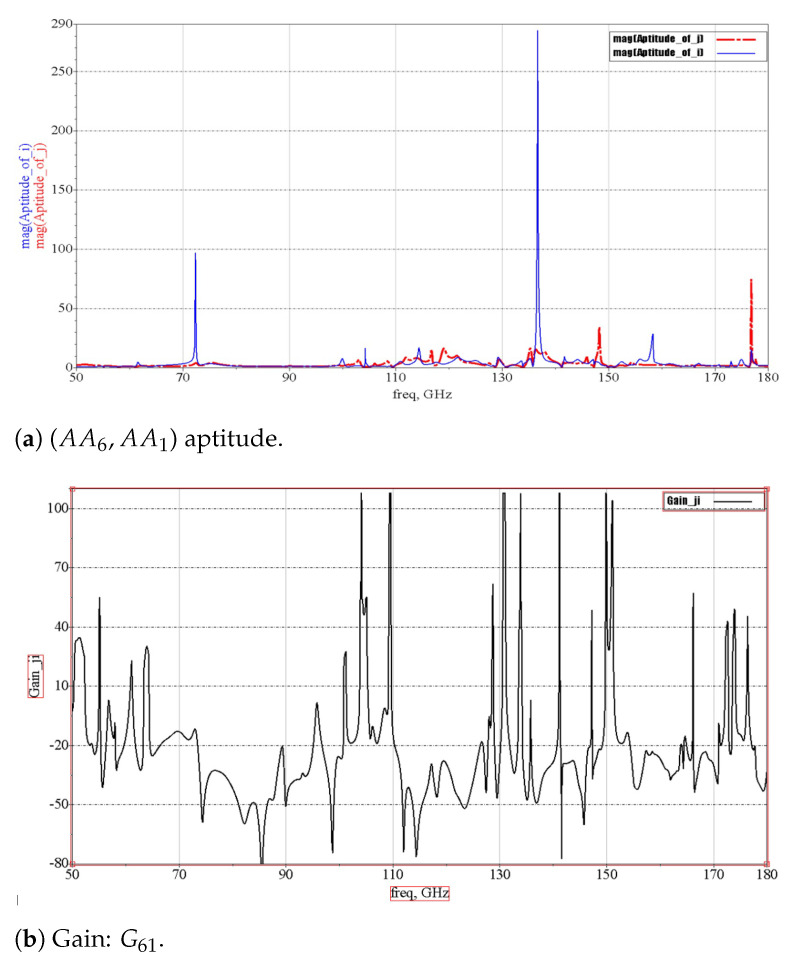
Transmission from $IP_{A1}$ to $IP_{A6}$.

The studied disposition is shown in Figure 13 as 4×4 IP-antennas ($IP_{A}$) in a square grid so 16 $IP_{A_{s}}$ are enumerated from 1 to 16. Each $IP_{Aj}$ can transmit to $IP_{Ai}$ on exclusive channels, chosen as having the optimum (available and highest) transmission gain. In the case that $IP_{Aj}$ would transmit data to $IP_{Ai}$, noted by $A_{ij}=$$\left(IP_{Ai},IP_{Aj}\right)$, we have to respect the above rules (Rule 1 to Rule 4) to deduce the right frequency set band $Bd_{ij}$ (channels $Ch_{ij}$) corresponding to the maximization of $G_{ij}$. Thus, we obtain a bijection between $Bd_{ij}$ and $A_{ij}$. Figure 14a,b illustrates the case of i=1 and j=6, and Figure 14a depicts AA6−AA1 (acceptance aptitudes of $IP-A_{6}$–$IP-A_{1}$, calculated by means of Equation (6). Figure 14b shows the gain $G_{61}$, calculated using Equation (5). We have represented the gain $G_{ji}$ from $IP_{Ai}$ to $IP_{Aj}$. The acceptance aptitude of $IP_{Ai}$ is the inverse of the linear $S_{ii}$ given by Equation (6):

(6) $$AA_{i}={\left(mag\left(S_{ii}\right)\right)}^{-1}$$

**Figure 15:**
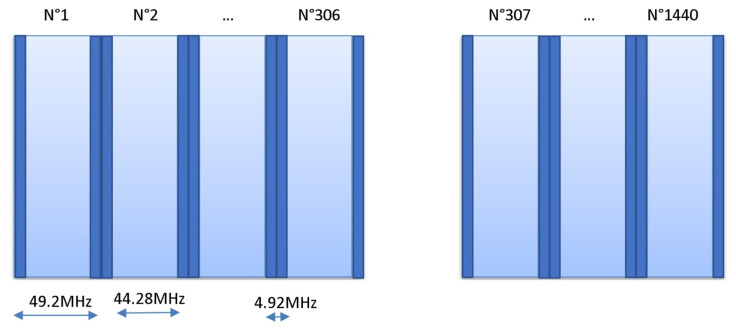
Structure of all sub-channels from 1 to 1440, considering 4.92 MHz inter-sub-channel gap.

In fact, such intersections should be well managed in roder to minimize interferences or coupling phenomena, which degrade on-chip antenna communications. An algorithm was developed to filter bijective spectral bands between $IP_{Ai}$ and its assigned spectral channels set. Thus, one chosen frequency cannot be used for two different $IP_{As}$ distinctly in the case of transmission and receiving signals. Assigning an exclusive set of channel frequencies for each $A_{ji}$ is actually the intersection of the highest $AA_{i}$, $AA_{j}$, and $G_{ji}$ values, where we omit the used channel frequencies of other $A_{ji}$. In the 4×4 $IP_{Ai}$ system, we distinguish 240 = 16 × 15 possible cases of $IP_{Ai}$, which involve at least 240 channel frequencies that we have to assign. On-chip IPs communicate simultaneously with each other, thanks to the unique channel assigned per wireless link. In our case, we have two bandwidths, respectively, of 15.06 GHz (78.32−63.26) and 55.8 GHz (157.0−101.2) around 70.86 GHz (15.06+55.8) that can be deployed to 240 $A_{ji}$, having six sub-channels of 49.2 MHz each (about 50 MHz). To achieve this aim, we subdivide 70,860/(240 × 6) about 70,860/1440 and obtain 49.2 MHz for each sub-channel. To avoid the interference issues, we do not use the tenth of 49.2, around 4.92 MHz, and we assign only six efficient sub-channels, having 44.28 MHz per couple $A_{ji}$, of which a tenth is about 4.92 MHz, used as a gap between two consecutive sub-channels, as depicted in Figure 15, where dark columns indicate frequencies intervals/gaps, which are not used. We identify each sub-channel by the mother band ($B_{1}$ or $B_{2}$), a number between 1 and 1440, an interval of operational frequencies having [f2, f4] limits centered in f3 and with two gaps (frequencies that are not used) [f1,f2[∪]f4,f5] as seen clearly in Table 4, then detailed in Table 5 for the case of $A_{61}$ sub-channels. The relation between frequency and sub-channel number ($NSCh_{ij}$) is presented in Equation (7) as follows:

(7) $$NSCh_{i}j=roundfloor\left(LSChN+\left(Fr_{i}j-BS_{p}\right)/0.0492\right)$$

where LSChN is the last sub-channel number of the preceding band, $Fr_{ij}$ is the depicted frequency from the gain $G_{ij}$ sorted in descending order, and $BS_{p}$ is the starting frequency of the current band number *p*. In our case, the antenna provides two bands, so that *p* is equal to 1 or 2. In this example, $Fr_{61}=153$ GHz, LSChN=306, p=2, and $BS_{2}=101.2$ GHz; this gives $NSCh_{61}=1360$.

The filling of Table 4 and the deduction of Table 5 is accomplished according to an efficient assignment of frequencies, according to Algorithm 1.


**Algorithm 1** Frequencies’ assignment algorithm per A*ij*
(Input parmeters: *n*, *m*) (here *n* = 16 and *m* = 6)
(Output: *Alloc*([1..(*n* ∗ (*n* − 1) ∗ *m*)]) (here *Alloc*([1..1440])

Initialization (*G*, *Fr*, *Alloc*, *qij*)REPEAT(a)FOR each *A*_ij_*qij* ← 0REPEAT*qij* ← *qij* + 1*NSCh* ← *round_f_loor* (*LSChN* + (*Fr*(*i*, *j*, *qij*) − BSp)/0.0492)UNTIL (Not(Alloc[NSCh]))

Alloc[NSCh].V←True



Alloc[NSCh].Tx←j



Alloc[NSCh].Rx←i



Alloc[NSCh].N←U

(b)ENDFOR(c)

U←U+1

UNTIL (U≥(m+1))


**Figure 16:**
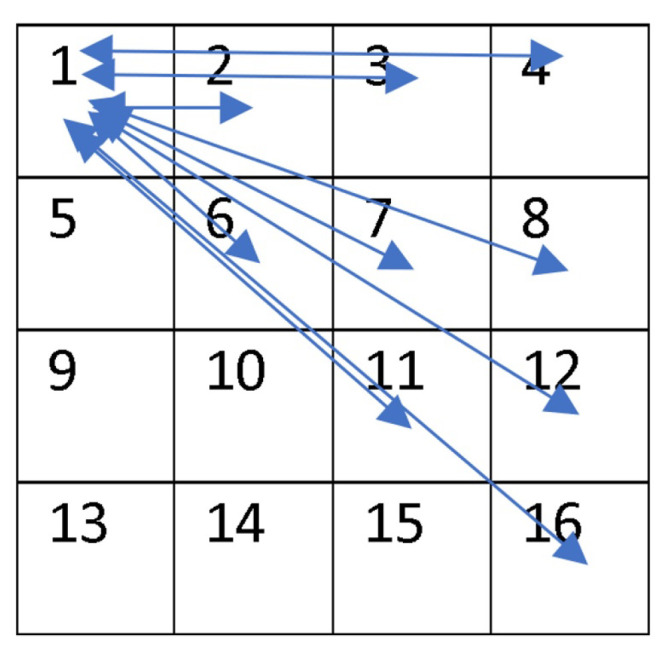
All possible distances inter $IP_{A_{i}}$: [4.24, 3.6, 3.16, 3.0, 2.82, 2.23, 2.0, 1.41, 1.0].

**Figure 17:**
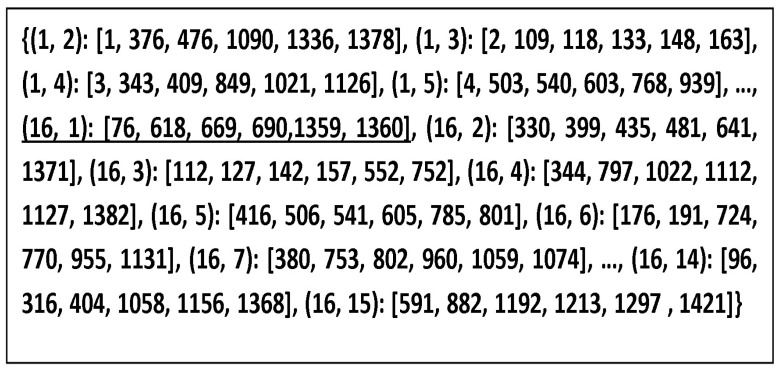
A sample of an assignment plan for the list of 240 $A_{ji}$ (here m=6) ($IP_{A_{j}}$, $IP_{A_{i}}$): $\left[Nsch_{1},...,Nsch_{6}\right]$. The 6 underlined numbers are the assigned frequencies to $IP_{16}$ to communicate with $IP_{1}$.

Where *U* is an integer counter of the sub-channel assignment number in {1, 2, 3, 4, 5, 6}; *G* is a set of $G_{ij}$ for each $A_{ij}$ deduced from Equation (5); Fr is a set Fr(i,j,qij) sorted in descending order for each (i,j) according to $G_{ij}$; and Alloc is an array of 1440 (sub-channels) structures composed of (*V*: Boolean;Tx,Rx, *N*: integer), where *V* indicates if the sub-channel is allocated, Tx is the number of the transmitting antenna ($IP_{Aj}$), and Rx is the number of the receiving antenna ($IP_{Ai}$), and *N* is order number of assigned sub-channels. The algorithm is optimized by considering Equation (5). According to this equation, the received power decreases considerably when the inter-antenna distance increases. The set of 240 $A_{ij}$ should be sorted in descending order by the distance $d_{ij}$ between $IP_{Ai}$ and $IP_{Aj}$, as presented in Figure 16. Actually, the number *m* of assigned sub-channels per $A_{ij}$ (here m=6) is an important parameter. When it is changed, we modify the sub-channel width SumBw/((n(n−1)m)), where SumBw=70.8 GHz and n is the number of antennas (16 = 4 × 4). However, whenever we execute Algorithm 1 we will obtain a new frequency plan per $A_{ij}$. A sample of an assignment plan resulting from the proposed Algorithm 1 is shown in Figure 17, where each of the 240 $A_{ij}$ antenna couples is assigned six exclusive sub-channels. This can help to hop frequencies and obtain easier FTDM access.

## 6. Conclusions

In this work, we have presented the design of an innovative millimeter-wave fractal antenna to enable on-chip pure wireless communications. The antenna operates efficiently in two wide bands, $B_{1}$ (63−78 GHz) and $B_{2}$ (101−157 GHz). The proposed antenna system-(4×4) showed satisfactory communication performance in terms of communication between many sub-channels, size, gain, polarization, and bandwidth. The antenna is practically qualified to be integrated on-chip.

We subdivided $B_{1}$ and $B_{2}$ into 1440 sub-channels, with 49.2 MHz as the width, and only 44.28 MHz were exploited and the 4.92 MHz band remained as a gap inter-sub-channel to eliminate the risk of interference between two consecutive sub-channels. We assigned exclusively to each antenna couple $A_{ij}=\left(A_{j},A_{i}\right)$*m* (six) sub-channels, sorted by their gain $G_{ij}$. Thus, the first sub-channel corresponds to the best quality and the sixth to the lowest quality. Hence, the wireless NoC provides requirements such as reliability, guaranteed bandwidth, and latency.

The proposed wireless NoC can efficiently improve performance. Moreover, the WiNoC should overcome limitations due to the increased likelihood of timing and data-errors, crosstalk, and environmental factors such as electromagnetic interference, which represent issues to confront and challenges to overcome in future works. As a first perspective, frequency hopping via time slot multiple access (FDMA/TDMA/FH−TDMA) may be an alternative solution, meaning that one chosen frequency could be used by two different $IP_{A}$s at different time slots. The second perspective is to assign not only one antenna but two radiators per IP to ensure full-duplex communication, with the first antenna used transmission and the second one used for receiving.

## Figures and Tables

**Table 1:** −10 dB frequency markers of NoChA.

Markers	Frequency (GHz)	$S_{11}$ (dB)
mi	Fi	S11
m1	63.26	−10.469
m2	76.25	−23.136
m3	78.32	−10.2016
m4	101.2	−10.469
m5	103.6	−21.630
m6	116.1	−47.364
m7	140.0	−42.129
m8	157.0	−9.839

**Table 2:** Antenna parameters.

F GHz	$E_{\max}$ (V)	$\Theta_{\max}\;\left(\right)$	$\Phi_{\max}\;\left(\right)$	$D_{\max}$ (dB)	$G_{\max}$ (dB)	Radiated (W)	Input (W)	η
77	0.264	162.000	245.000	5.860	−3.240	3.018 $\times \;10^{-4}$	0.002	0.123
106	0.168	141.000	62.000	4.683	−7.186	1.606 $\times \;10^{-4}$	0.002	0.065
113	0.179	141.000	58.000	5.921	−6.679	1.369 $\times \;10^{-4}$	0.002	0.055
123	0.149	129.000	269.000	2.869	−8.172	1.291 $\times \;10^{-4}$	0.002	0.079
141	0.198	176.000	39.000	4.883	−5.795	2.11 $\times \;10^{-4}$	0.002	0.086

**Table 3:** Performance comparison of NoChA.

Reference	Size (mm^2^)	Bw (GHz)	Gain (dB)	Frequency (GHz)	Antenna Type
[34]	0.023	51–66	−26.8	60	not reported
[35]	1.2	318–370	Not reported	344	patch
[36]	10.54	55–65	−10.6	60	Vivaldi
[37]	2.85	305–375	1.85	340	differential rectangular patch antenna
[38]	Not reported	26–40	1.69	60	dipole
[39]	1.6	63.5–68.5	−1.4	not reported	*TSVA*
This	0.824	B1(63 to 78)	−3.24	77	patch
work		B2(110 to 157)	−5.795	140	

**Table 4:** Antenna frequency assignment.

		Band_1 (63.26–78.32) GHZ							Band_2 (101.2–157.0) GHz									
		Sub-Channels N°																
N°	A*_i,j_*	**1**	**2**	**3**	**..**	**76**	**..**	**306**	**307**	**618**	**..**	**690**	**..**	**1359**	**1360**	**..**	**..**	**1440**
**1**	A_1,2_																	
**2**	A_1,3_																	
**..**	**..**	**..**	**..**	**..**	**..**	**..**	**..**	**..**	**..**	**..**	**..**	**..**	**..**	**..**	**..**	**..**	**..**	**..**
**76**	A_6,1_					**66.9**				**116.5**		**120**		**152.9**	**153**			
**..**	**..**	**..**	**..**	**..**	**..**	**..**	**..**	**..**	**..**	**..**	**..**	**..**	**..**	**..**	**..**	**..**	**..**	**..**
**240**	A_16,15_																	

**Table 5:** Used sub-channels [*f*2, *f*4]; gaps [*f*1, *f*2] and [*f*4, *f*5]—used for *A*_61_ communication.

Subch N°	*f*1 (Hz)	*f*2 (Hz)	*f*3 (Hz)	*f*4 (Hz)	*f*5 (Hz)
76	66 950624975	66 953085391	66 975229141	66 997372891	66 999833308
618	116 501541461	116 504001877	116 526145627	116 548289377	116 550749794
669	119 011166444	119 013626860	119 035770610	119 057914360	119 060374777
690	120 044541437	120 047001853	120 069145603	120 091289353	120 093749770
1359	152 964916214	152 967376630	152 989520380	153 011664130	153 014124547
1360	153 014124547	153 016584963	153 038728713	153 060872463	153 063332880

**Figure A1:**
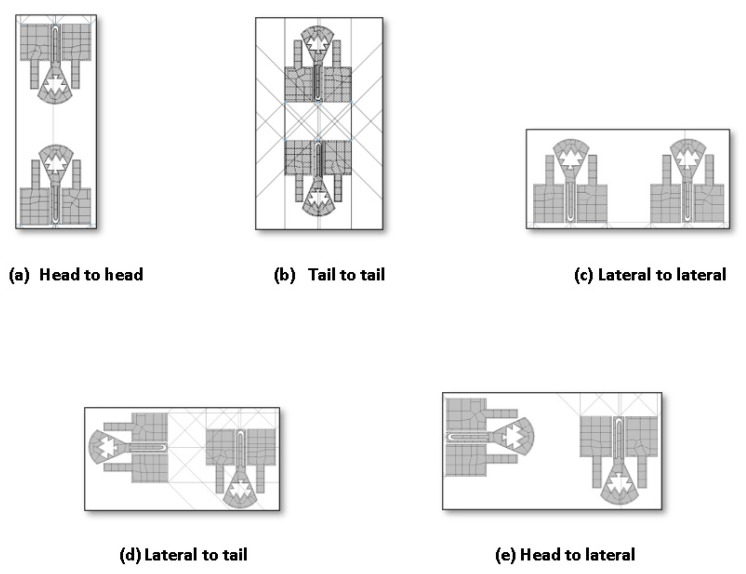
Five possible dual bi-NochA dispositions.

**Figure A2:**
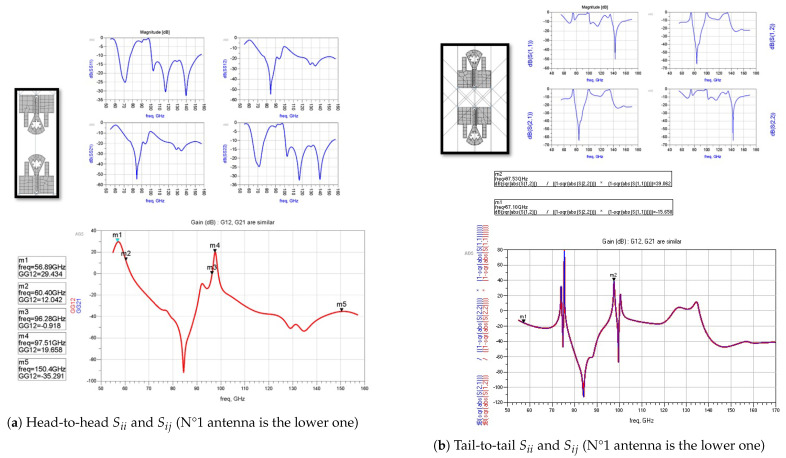
Head -to-head and tail-to-tail cases.

**Figure A3:**
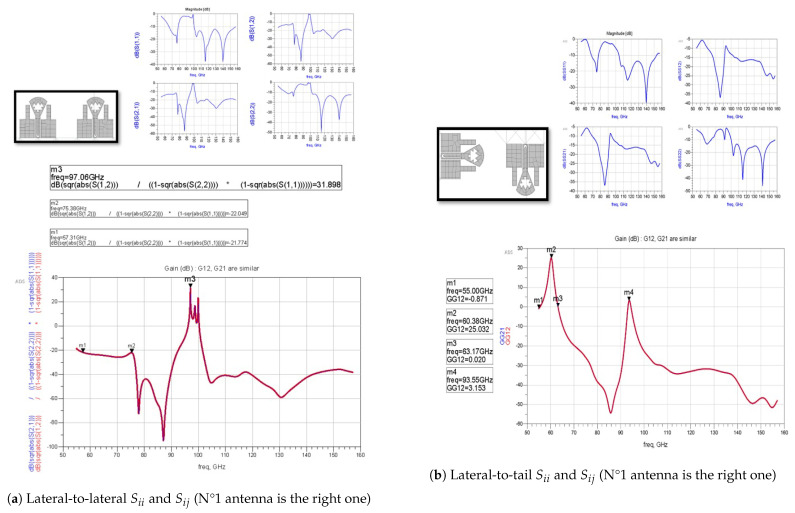
Lateral -to-lateral and lateral-to-tail cases.

**Figure A4:**
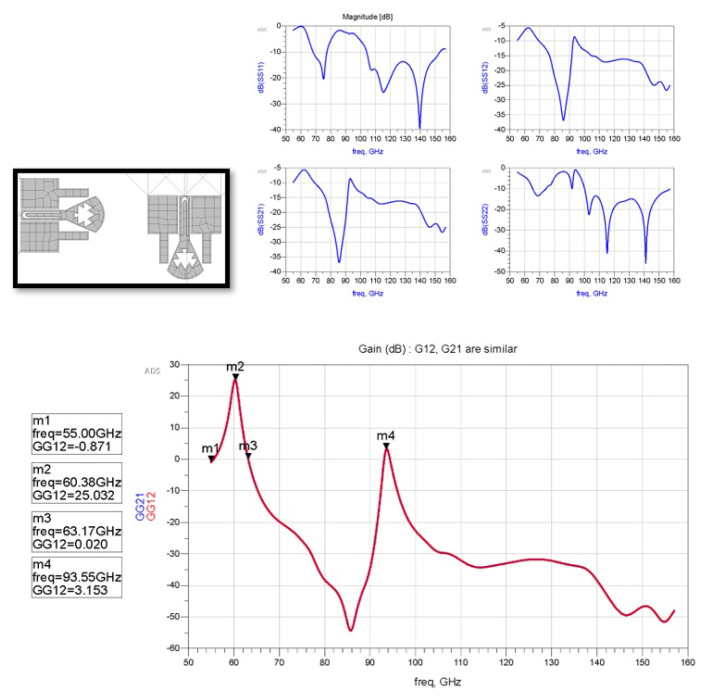
Head-to-lateral $S_{ii}$ and $S_{ij}$ (N°1 antenna is the left one).

**Figure A5:**
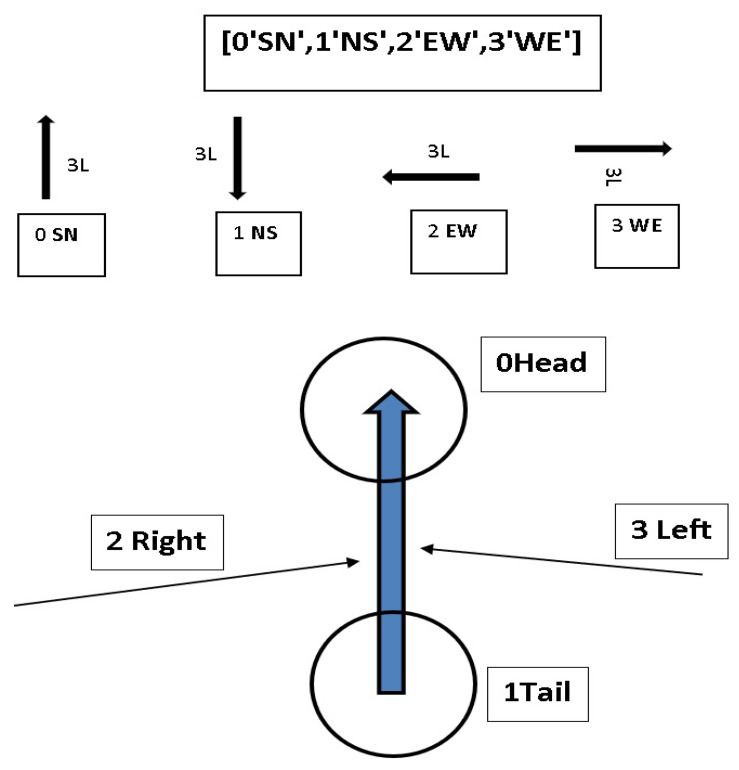
4 different cases coded from 0 to 3, S-south, N-north, E-east and W-west.

**Figure A6:**
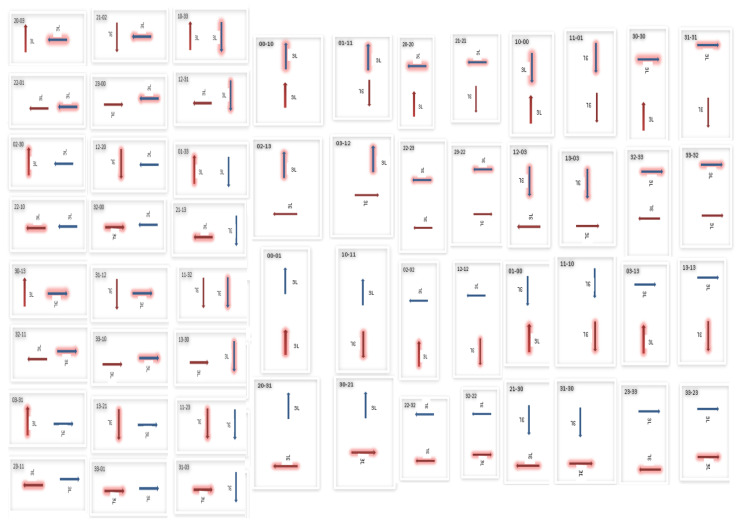
All the possible studied cases corresponding to Table A2.

## Abbreviations

The following abbreviations are used in this manuscript:

bi-WB	Bi-Wide-Band
FBR	fractional frequency band ratio
FBW	frequency band width (in percentage)
FDMA	Frequency-Division Multiple Access
FH-TDMA	Frequency Hopping TDMA
FS	Frequency-Selective Surface
IP	Intellectual Propriety
MPSoC	Multi-Processor SoC
NoC	Network on Chip
Qos	Quality of service
RFIC	Radio-Frequency Integrated Circuit
SoC	System on Chip
SOI	Silicon as innsulator
TDMA	Time-Division Multiple Access
UWB	Ultra-Wide-Band
WNoC	Wireless NoC

## Appendix A. Antenna Disposition Study

After the dual disposition study, we simulated different dual dispositions of our antenna *NoChA*, as shown in Figure A1; the corresponding results are presented in Figure A2, Figure A3, Figure A4. These figures represent ($S_{ii}$, $S_{ij}$) and gains ($G_{12}$, $G_{21}$).

### Appendix A.1. Antenna Disposition Weights

We note that if two IP antennas ($IP_{A1}$, $IP_{A2}$) communicate, their dispositions affects the transmission quality. Therefore, we have ensured that the weights correspond to each transmitting disposition, as shown in Figure A1, and then in Table A1. The best gain was observed in the case of a head-to-head system (Figure A2a), then in the head-to-lateral case (Figure A4), then in the case of lateral-to-lateral disposition (Figure A3a). The worst transmission cases were the tail-to-tail case (Figure A2b), then the lateral-to-tail case (Figure A3b), and the least worst case was the head-to-tail arrangement. The tail of the $IP_{A}$ is the half-antenna region where feeding, ports, pins, and weldings can appear, as in the bottom of Figure 3c.

**Table A1:** Tx/Rx Dispositions and their quality weights, example 1 (head-to-tail), coded 01, has a weight of 30 (second line); example 2 ($Left_{Lateral}$ to head), coded 30, has a weight of 40 (13th line).

[0:H, 1:T, 2:R, 3:L]	Quality Weight
00	100
01	30
02	60
03	60
10	2
11	1
12	3
13	3
20	40
21	10
22	30
23	30
30	40
31	10
32	30
33	30

### Appendix A.2. Antenna Disposition Cases

The (n∗m) disposition of antennas is always presented in a grid array of *n* lines and *m* columns, but questions remain regarding how to sow these $IP_{A}$s on the grid and which disposition is the best in terms of guaranteeing the optimum gain transmission of each antenna couple $A_{ij}$ = ($A_{i}$, $A_{j}$). A random searching algorithm was used to answer these questions. Thus, regardless of the value of *n* and *m* (≥2), we can obtain the best disposition, while maximizing a function weight for each situation. For example, if *n* equals *m*, which is 4 (the case presented in this paper), the best arrangement is that shown in Figure 13.

For this purpose, we coded the different cases [0, 1, 2, 3] corresponding to S—south, N—north, E—east, and W—west, as shown in Figure A5. Then, we listed all the possible arrangements of dual cases. $IP_{A}$Tx can be placed on or under the $IP_{A}$Rx, and it can also be placed beside the $IP_{A}$Rx from the right or from the left. All the cases (16 × 4 = 64) are illustrated in Table A2. The first column lists all the possible dispositions/codes. The second and the third column are devoted to horizontal dispositions. The forth and the fifth columns concern vertical dispositions. Hence, each disposition is described as being horizontal or vertical. For horizontal cases, transmission can occur from left to right → (coded 0) or from right to left ← (coded 1). For vertical cases, transmission can occur from down to up ↑ (coded 2) or from up to down ↓ (coded 3). In Figure A6, many cases are studied in both a horizontal and a vertical way.

**Table A2:** All possible disposition cases.

	Horizontal	Horizontal	Vertical	Vertical
**[0:SN, 1:NS, 2:EW, 3:WE]**	**0:** →	**1:** ←	**2:** ↑	**3:** ↓
00	32	23	01	10
01	33	22	00	11
02	30	21	02	13
03	31	20	03	12
10	22	33	11	10
11	23	32	10	01
12	20	31	12	03
13	21	30	13	02
20	21	03	31	20
21	13	02	30	21
22	10	01	32	23
23	31	00	33	22
30	02	13	21	30
31	03	12	20	31
32	00	11	22	33
33	01	10	23	32

### Appendix A.3. Investigation of Cases

In Figure 13, when the first $IP_{A1}$ radiates towards the 16th $IP_{A16}$, we mention the distance between them as $d_{1,16}=4.24$ mm (Figure 16) and the maximum number of crossed antennas as Crossed(1,16)=4 when a simple line joins them. These two parameters affect the involved weight $Wg_{1,16}$. We consider two distances: the first is the horizontal $L_{1,16}$ with a head-to-head link (00, $weight_{H}=100$), the second is the vertical $h_{1,16}$ with a lateral-to-lateral link (right to right: 22, $weight_{V}=30$). Therefore, $Wg_{1,16}$ is expressed as follows in Equation (A1):

(A1) $$Wg_{1,16}=W_{1,16}\left(d_{1,16},\left(L_{1,16},weight_{H}\left(00\right)\right),\left(h_{1,16},weight_{V}\left(22\right)\right),Crossed\left(1,16\right)\right).$$

For each (i,j), $Wg_{i,j}$ is proportional to $weight_{H}$ and $weight_{V}$, which depend upon Table A1 and Table A2. However, we have deduced four other parameters: ($d_{i,j},L_{i,j},h_{i,j},andCrossed\left(i,j\right)$), which are inversely proportional to $Wg_{i,j}$.

Eventually, we maximized $\sum_{i=1}^{n}\sum_{i=1}^{m}Wg_{i,j}$, and we chose the best corresponding arrangement. A chosen case with any *n* or *m* can be studied using our method. The proposal is that each $IP_{A}$ noted $A_{i}$ transmits to $A_{j}$ using its own set of channels (Figure 17), ensuring that there are no more interferences and fewer problems of time division access. Each antenna couple $A_{ij}$ ($Tx_{i}$, $Rx_{j}$) will have specific frequencies with which to communicate. Consequently, the detected frequency signal (x(f)) will help to obtain the right couple $A_{ij}$ and vice-versa. To conclude, the target of obtaining a bijection between $A_{ij}$ and its set of frequencies was achieved thanks to our proposed NoChA antenna.

## References

[1] Karim R., Iftikhar A., Ijaz B., Ben Mabrouk I. (2019). The Potentials, Challenges, and Future Directions of On-Chip-Antennas for Emerging Wireless Applications—A Comprehensive Survey. IEEE Access.

[2] Balti M., Abderrazzak J. (2021). Performance survey of classic and Optic network on chip. IET Circuits-Devices-Syst..

[3] Gutierrez F. (2017). Design of a Wideband Antenna for Wireless Network-On-Chip in Multimedia Applications. J. Low Power Electron. Appl..

[4] Devanathana M., Ranganathanb V., Sivakumarc P. (2020). Congestion-aware wireless network-on-chip for high-speed communication. Autom. J. Control. Meas. Electron. Comput. Commun..

[5] Abbosh A.M., Bialkowski M.E. (2008). Design of Ultrawideband Planar Monopole Antennas of Circular and Elliptical Shape. IEEE Trans. Antennas Propag..

[6] Angelopoulos E.S., Anastopoulos A.Z., Kaklamani D.I., Alexandridis A.A., Lazarakis F., Dangakis K. (2006). Circular and Elliptical CPW-Fed Slot and Microstrip-Fed Antennas for Ultrawideband Applications. IEEE Antennas Wirel. Propag. Lett..

[7] Al-Gburi A.J.A., Ibrahim I.M., Zakaria Z., Abdulhameed M.K., Saeidi T. (2021). Enhancing Gain for UWB Antennas Using FSS: A Systematic Review. Mathematics.

[8] Kumar O.P., Kumar P., Ali T. (2022). A Compact Dual-Band Notched UWB Antenna for Wireless Applications. Micromachines.

[9] Karkar A., Mak T., Tong K.F., Yakovlev A. (2016). A Survey of Emerging Interconnects for On-Chip Efficient Multicast and Broadcast in Many-Cores. IEEE Circuits Syst. Mag..

[10] Marculescu R., Ogras U.Y. (2009). Outstanding research problems in NoC design: System, micro-architecture, and circuit perspectives. IEEE Trans.-Comput.-Aided Des. Integr. Circuits Syst..

[11] Rusli M.S., Lit A., Marsono M.N., Palesi M. (2017). Adaptive Packet Relocator inWireless Network-on-Chip (WiNoC). Modeling, Design and Simulation of Systems.

[12] Mineo A., Palesi M., Ascia G., Catania V. An adaptive transmitting power technique for energy efficient mm-wave wireless NoCs. Proceedings of the 2014 Design, Automation Test in Europe Conference Exhibition (DATE).

[13] Rusli M.S., Mineo A., Palesi M., Ascia G., Catania V., Marsono M. A closed loop control based power manager for winoc architectures. Proceedings of the MES ’14: International Workshop on Manycore Embedded Systems.

[14] Lit A., Rusli M., Marsono M. (2019). Comparative performance evaluation of routing algorithm and topology size for wireless network-on-chip. Bull. Electr. Eng. Inform..

[15] Zhao D., Wang Y., Li J., Kikkawa T. Design of multi-channel wireless NoC to improve on-chip communication capacity. Proceedings of the NOCS ’11: Fifth ACM/IEEE International Symposium on Networks-on-Chip.

[16] Ortiz Sosa J., Sentieys O., Roland C. Adaptive Transceiver for Wireless NoC to Enhance Multicast/Unicast Communication Scenarios. Proceedings of the 2019 IEEE Computer Society Annual Symposium on VLSI (ISVLSI).

[17] Deb S., Chang K., Ganguly A., Yu X., Teuscher C., Pande P., Heo D., Belzer B. Design of an efficient NoC architecture using millimeter-wave wireless links. Proceedings of the International Symposium on Quality Electronic Design, ISQED.

[18] Wang Q., Ouyang Y., Lu Y., Liang H., Zhu D. (2021). Neural Network-based Online Fault Diagnosis in Wireless-NoC Systems. J. Electron. Test..

[19] Mondal H.K., Kaushik S., Gade S.H., Deb S. Energy-Efficient Transceiver for Wireless NoC. Proceedings of the 2017 30th International Conference on VLSI Design and 2017 16th International Conference on Embedded Systems (VLSID).

[20] Xia H., Bertoni H., Maciel L., Lindsay-Stewart A., Rowe R. (1993). Radio propagation characteristics for line-of-sight microcellular and personal communications. IEEE Trans. Antennas Propag..

[21] Feuerstein M., Blackard K., Rappaport T., Seidel S., Xia H. (1994). Path loss, delay spread, and outage models as functions of antenna height for microcellular system design. IEEE Trans. Veh. Technol..

[22] Sommer C., Joerer S., Dressler F. On the applicability of Two-Ray path loss models for vehicular network simulation. Proceedings of the 2012 IEEE Vehicular Networking Conference (VNC).

[23] Karedal J., Czink N., Paier A., Tufvesson F., Molisch A.F. (2011). Path Loss Modeling for Vehicle-to-Vehicle Communications. IEEE Trans. Veh. Technol..

[24] Kunisch J., Pamp J. Wideband Car-to-Car Radio Channel Measurements and Model at 5.9 GHz. Proceedings of the 2008 IEEE 68th Vehicular Technology Conference.

[25] Zöchmann E., Lerch M., Caban S., Mecklenbräuker C., Mecklenbrauker C., Rupp M. Directional evaluation of receive power, Rician K-factor and RMS delay spread obtained from power measurements of 60 GHz indoor channels. Proceedings of the 2016 IEEE-APS Topical Conference on Antennas and Propagation in Wireless Communications (APWC).

[26] Friis H. (1946). A Note on a Simple Transmission Formula. Proc. IRE.

[27] Narde R.S., Mansoor N., Ganguly A., Venkataraman J. On-chip antennas for inter-chip wireless interconnections: Challenges and opportunities. Proceedings of the 12th European Conference on Antennas and Propagation (EuCAP 2018).

[28] Gade S.H., Ram S.S., Deb S. (2019). Millimeter wave wireless interconnects in deep submicron chips: Challenges and opportunities. Integration.

[29] Gaha H., Balti M. Design of on-chip fractal antenna for wireless NoC. Proceedings of the International Conference on Electrical, Computer and Energy Technologies (ICECET).

[30] Saponara S., Neri B. (2019). System-level analysis for integrated power amplifier design in mmWave consumer wireless communications. Lecture Notes in Electrical Engineering.

[31] Saponara S., Neri B. mm-wave integrated wireless transceivers: Enabling technology for high bandwidth connections in IoT. Proceedings of the 2015 IEEE 2nd World Forum on Internet of Things (WF-IoT).

[32] Xi T., Huang S., Guo S., Gui P., Zhang J., Choi W., Huang D., Kenneth K.O., Fan Y. A new compact high-efficiency mmWave power amplifier in 65 nm CMOS process. Proceedings of the 2015 IEEE MTT-S International Microwave Symposium.

[33] Neve C.R. (2012). Small- and Large-Signal Characterization of Trap-Rich HR-Si/HR-SOI Wafers for SoC Applications. Ph.D. Thesis.

[34] Laha S., Sidhu S.K. Feasibility of Full Duplex Communication for Wireless Network on Chips with OOK Modulation. Proceedings of the IEEE 21st Annual Wireless and Microwave Technology Conference (WAMICON).

[35] Jalili H., Momeni O. 17.10 A 318-to-370GHz Standing-Wave 2D Phased Array in 0.13 µm BiCMOS. Proceedings of the 2017 IEEE International Solid-State Circuits Conference (ISSCC).

[36] Calò G., Alam B., Bellanca G., Fuschini F., Barbiroli M., Tralli V., Bassi P., Stomeo T., Bozzetti M., Kaplan A.E. Dielectric and Plasmonic Vivaldi Antennas for On-Chip Wireless Communication. Proceedings of the 2019 21st International Conference on Transparent Optical Networks (ICTON).

[37] Al-Eryani J., Knapp H., Kammerer J., Aufinger K., Li H., Maurer L. (2018). Fully Integrated Single-Chip 305–375-GHz Transceiver With On-Chip Antennas in SiGe BiCMOS. IEEE Trans. Terahertz Sci. Technol..

[38] Masri I.E., Le Gouguec T., Martin P.M., Allanic R., Quendo C. Integrated dipole antennas and propagation channel on silicon in Ka band for WiNoC applications. Proceedings of the 2018 IEEE 22nd Workshop on Signal and Power Integrity (SPI).

[39] Pano V., Tekin I., Yilmaz I., Liu Y., Dandekar K.R., Taskin B. (2020). TSV Antennas for Multi-Band Wireless Communication. IEEE J. Emerg. Sel. Top. Circuits Syst..

